# Navigating Nonlinear Pathways: Challenges and Opportunities for Diversity, Equity, and Inclusion Leaders in Academic Emergency Medicine

**DOI:** 10.1016/j.acepjo.2025.100060

**Published:** 2025-02-14

**Authors:** Melanie F. Molina, Annika Bhananker, Beatrice Torres, Dalia Owda, Edgardo Ordoñez, Anita N. Chary

**Affiliations:** 1 Department of Emergency Medicine, University of California, San Francisco, California, USA; 2 Center for Innovations in Quality Effectiveness and Safety, Michael E. DeBakey United States Department of Veterans Affairs Medical Center, Houston, Texas, USA; 3 Department of Management, Policy, and Community Health, School of Public Health, University of Texas Health Science Center, Houston, Texas, USA; 4 Department of Emergency Medicine, Henry Ford Health System, Detroit, Michigan, USA; 5 Department of Medicine, Baylor College of Medicine, Houston, Texas, USA; 6 Henry J.N. Taub Department of Emergency Medicine, Baylor College of Medicine, Houston, Texas, USA

**Keywords:** diversity, equity, inclusion, emergency medicine

## Abstract

**Objectives:**

Diversity, equity, and inclusion (DEI) leadership roles have grown in academic emergency medicine (EM). We sought to elucidate specific pathways to DEI leadership roles among current DEI leaders in academic EM.

**Methods:**

From March to May 2023, we conducted semistructured, qualitative interviews with DEI leaders in academic EM across 5 US regions to investigate their pathways to leadership. Participants were recruited via email using Accreditation Council for Graduate Medical Education-accredited EM residency websites and the Academy for Diversity and Inclusion in EM. After recording and transcribing the interviews, we used an inductive approach to identify major themes.

**Results:**

Of 56 DEI leaders contacted, 25 agreed to participate, and 21 were interviewed. The median (range) interview duration was 34 (25-63) minutes. Leadership titles included directors, chairs, vice chairs, committee chairs, chiefs, advisors, and deans. Three major themes emerged: (1) nonlinear pathways—participants reached DEI roles through informal assumption, volunteering, or self-creation, often without initial aspiration or compensation; (2) undefined roles and expectations—roles and responsibilities were often determined by leaders themselves, with advantages and disadvantages; (3) variable perceived value in promotions—participants felt DEI efforts were frequently undervalued in academic promotion, with mentorship highlighted as crucial for translating DEI activities into academic achievements.

**Conclusion:**

Our study provides important insights not only into the pathways to DEI leadership among current leaders in academic EM but also into the challenges and opportunities DEI leaders perceive when navigating roles, responsibilities, and academic promotion.

**The Bottom Line**

This study explored how academic emergency medicine (EM) faculty members assume formal diversity, equity, and inclusion (DEI) leadership roles. It found that pathways to these roles are often nonlinear, with most assuming roles informally or being volunteered by administrators and others creating the role themselves. DEI roles are frequently undefined and under-resourced, making it challenging for faculty to balance DEI work with other scholarly activities necessary for promotion. The study highlights the need for formalized pathways to DEI leadership, adequate resourcing, and recognition of DEI efforts in promotion processes to attract and support DEI leaders in academic EM.

## 1 Introduction

### 1.1 Background and Importance

Diversity, equity, and inclusion (DEI) are important principles in emergency departments (EDs), which often serve racially and ethnically diverse populations.^1^ Diverse perspectives lead to enhanced problem-solving,^2^ and diversity among physicians is associated with higher patient satisfaction,^3^ greater illness comprehension,^4^ and improved medication adherence^5^ among minoritized populations.

Recently, many academic EDs have created leadership positions for faculty to spearhead DEI efforts.^6^ A 2022 survey of 79 EDs found that 74.7% had appointed a DEI leader. Most DEI leaders were non-White and nontenure track, and less than half received compensation for their efforts.^6^

### 1.2 Goals of This Investigation

As part of a larger study of academic emergency medicine (EM) faculty experiences in DEI leadership roles, we sought to elucidate specific pathways to DEI leadership roles among current DEI leaders in academic EM.

## 2 Methods

### 2.1 Study Design, Setting, and Selection of Participants

We performed a descriptive qualitative study. As part of our recruitment process, we reviewed all US Accreditation Council for Graduate Medical Education-accredited EM residency websites for DEI leadership positions to develop a list of potential participants, supplemented with institutional or national DEI leaders known to executive committee members of the Academy for Diversity and Inclusion in EM. Given the geographic variation in DEI climates at higher educational institutions, we purposefully sampled at least 2 to 3 participants from 5 geographic regions of the US: Northeast, Southwest, West, Southeast, and Midwest. After being recruited via email (Supplementary Appendix 1), participants consented orally and received $50 compensation once they completed a virtual interview. The Baylor College of Medicine Institutional Review Board provided ethics approval. We followed the Consolidated Criteria for Reporting Qualitative Research (COREQ) reporting guidelines (Supplementary Appendix 2).^7^

### 2.2 Data Collection

Based on prior literature providing foundational insights into responsibilities, efforts, and professional impacts related to diversity efforts in academic medicine,^8^, ^9^, ^10^ 2 team members (E.O. and A.N.C.) drafted an interview guide to assess factors that led to participants assuming DEI leadership roles. The initial guide was refined based on 4 pilot interviews with EM faculty members and feedback from all investigators. The final guide included participant demographic variables, such as self-reported race and ethnicity and years of experience (Supplementary Appendix 3). Between March and May 2023, 3 research assistants—who identified as women from racial or ethnic minority groups and who had no prior relationships with participants (A.B., B.T., and V.R.)—conducted one-on-one, semistructured interviews by videoconference. The research assistants were trained in interviewing by a qualitative expert with a PhD in anthropology (A.N.C.). They prioritized open-ended questions to gather data grounded in the participants’ own experiences. All interviews were audio recorded and professionally transcribed. We did not return transcripts to participants but reviewed them for accuracy and deidentification.

### 2.3 Data Analyses

The study team used an inductive approach to develop a codebook based on a preliminary review of transcripts.^11^ Two researchers (M.F.M. and A.B.) then independently coded each transcript using Excel (Microsoft 2024, version 16.92). The study team resolved coding discrepancies and identified emerging themes via consensus to mitigate bias. Analysis occurred after all interviews were completed, and participants did not provide feedback on the resulting themes. Thematic saturation was reached by 12 interviews.

## 3 Results

Of 56 DEI leaders in academic EM contacted, 25 agreed to participate, and 21 were interviewed (4 were unable to complete an interview due to limited availability). The median (range) interview duration was 34 (25-63) minutes. Table 1 summarizes participant demographics. Leadership titles included directors, chairs, vice chairs, committee chairs, chiefs, advisors, and deans. We identified 3 major themes related to pathways to DEI leadership roles, as well as challenges and opportunities for DEI leaders: (1) nonlinear pathways, (2) undefined roles and expectations, and (3) variable perceived value toward promotion. Table 2 provides quotes illustrative of these 3 themes.

**Table 1:** Characteristics of diversity, equity, and inclusion leaders in academic emergency medicine.

Characteristic	Participants, N (%)
Participants (N = 21)	
Academic rank	
Assistant professor	8 (38)
Associate professor	8 (38)
Professor	5 (24)
Self-reported race	
Asian	2 (10)
Black	8 (38)
Multiracial	3 (14)
Other^a^	2 (10)
White	6 (29)
Self-reported ethnicity	
Hispanic	2 (10)
Sex	
Man	9 (43)
Woman	12 (57)
LGBTQIA+	6 (29)
Region	
Midwest	3 (14)
Northeast	5 (24)
Southeast	5 (24)
Southwest	5 (24)
West	3 (14)

LGBTQIA+, lesbian, gay, bisexual, transgender, queer or questioning, intersex, asexual, and any other identities not specifically covered by letters in this acronym.

a “Other” included Jewish and Caribbean Americans.

**Table 2:** Key themes and representative brief quotations from DEI leaders in academic emergency medicine on their pathways to DEI leadership roles.

Theme and subtheme	Quotation
Nonlinear pathways	
Created the position	“I actually put together the initial proposal for the job that I right now hold. So I created a job, I created a job title, you know, like, a mission statement goals, objectives, and then put it forth to the chair of my department to, you know, was in favor because we did that.” -Participant 1003 (a minoritized individual with departmental and national roles).
Informally assumed the position	“There was no one else there to do the work. And this is stuff that I’m passionate about. This is work that I’m passionate about.” -Participant 1006 (a minoritized individual with departmental and national roles).
Volunteered by leadership	“I was pushed forward. And I do feel like it was because (they) were trying to diversify this committee and that committee...‘I think you’ll be great at it,’– which they will tell you until you end up being signed up for way too many things.” -Participant 1014 (a minoritized individual with departmental and national roles).
Undefined roles and expectations	“I essentially oversee all efforts with a number of different things in DEI. In particular, the 5 strategic goals or priorities, I created them in different areas, such as people, patient care, outreach, culture and climate, professional development, and things of that nature. And then within that there's subcommittees that are broken up, essentially, they cover different strategic goals. Also, you might have different topics. So you can figure out how to do certain things and navigate projects.” -Participant 1008 (a minoritized individual with a departmental role).
	“Responsibilities have mostly been whatever I've decided to make them so if I want like we've put on some stuff about like mentoring, like we've done workshops, like on the institutional level that I helped put together with some of the students' stuff like as the faculty advisor for SMNA. You kind of prioritize the students and what their interest is and how you can best help them.” -Participant 1002 (a minoritized individual with departmental, institutional, and national roles).
Variable perceived value toward promotion	“I believe that some avenues to promotion are a bit more clear and like, aligned, you know, with some of the missions that people actually value. Social media and DEI-related things are a bit new in this space. And I don't think that it gets as much recognition for a lot of the hard work, especially when you think of like community service, which probably doesn't have compensation component. So people may not see the value of that. I think that it would be interesting if there was a track that could lead to promotion, when it comes to like maybe community engagement or service and how much we're contributing in that way.” -Participant 1012 (a minoritized individual with departmental, institutional, and national roles).
	“Even though I had, you know, over 100 national presentations and, you know, had multiple publications (on LGBTQ+ health), the person who’s in charge of the promotion committee basically told me, ‘Well, you can’t use any of that because that’s not academic.’ You know, and that was clearly very wrong.” -Participant 1019 (a nonminoritized individual with institutional and national roles).

DEI, diversity, equity, and inclusion; LGBTQ+, lesbian, gay, bisexual, transgender, queer/questioning; SMNA, Student National Medical Association.

### 3.1 Theme 1: Nonlinear Pathways

Participants described 1 of 3 pathways to DEI leadership roles: informally assuming, being volunteered for, or creating the position. Fifteen participants either informally assumed or were volunteered for their positions by administrative leaders. These participants usually had not initially aspired to a DEI leadership role:

“I was probably voluntold…I didn’t seek their role…It was more handed to me.” (Participant 1002, a minoritized individual with departmental, institutional, and national roles).

One participant described having performed uncompensated DEI work for years before being offered a role with buydown in the wake of the George Floyd murder and at the impetus of the School of Medicine:

“…part to deal with what happened with George Floyd…they (the School of Medicine) want every clinical department…to say (they) have a clinical lead for (DEI work)…my chair knew that I’ve been doing this and asked me if I’d be interested in serving this way. And I said, Yeah, sure. (Previously) I wasn’t really getting any sort of buydown or compensation, so to speak, I was just like doing it and everything else, whereas now I do get time for this.” (Participant 1008, a minoritized individual with a departmental role).

Six participants created their own leadership positions. They reported feeling a lack of action without their initiative, describing their efforts as necessary to establish a DEI presence in their department/institution. One of these participants described strategically building their DEI role into other departmental or institutional activities to circumvent a lack of departmental support:

“So I created it (DEI leadership role). We had, and we continue to not have support from our department in this space. So because of that, I kind of found a way around it, and created our DEI committee in a way that it’s a subset of some other areas for the department to allow me to do work in this space.” (Participant 1011, a nonminoritized individual with departmental and institutional roles).

### 3.2 Theme 2: Undefined Roles and Expectations

Participants reported a lack of definition surrounding their roles and expectations. This was pervasive among participants who occupied traditional director or vice chair roles and those who occupied nontraditional roles (ie, committee chair and departmental DEI champion). One participant detailed how the lack of clear expectations, albeit with a formal DEI leadership title, translated into mismatched resourcing:

“People understand roles (such as) director, vice chair…(DEI) roles themselves are very poorly defined. And so, in being poorly defined, they may not necessarily be as resourced. I did not feel the amount of time I got reimbursed was equivalent to the amount of time that went into the role. And the expectations of the administration were still kind of nebulous. I guess the easiest way to put it is: having somebody in that title, a lot of people who don’t know any better just kind of assume that means everything’s better. And it’s far from it. Especially if without guidance, without resources, without much of anything but a title and a little bit of time.” (Participant 1001, a minoritized individual with departmental and institutional roles).

Another participant did not perceive the lack of a formalized DEI role within their department to be a hindrance to DEI efforts but rather allowed for broad integration of DEI into departmental initiatives:

“There’s no formal DEI role in terms of a leadership position in the department. We all collectively think about that in our work. For example, when we’re creating scholarships, we think about DEI so we created a specific fund for summer students. Or similarly, when there are faculty recruitment sort of meetings, we think about DEI in that sort of space.” (Participant 1005, a nonminoritized individual with institutional and national roles).

Although 5 participants held formal educational or faculty development positions with some defined mentoring and advising responsibilities, most participants described leading efforts of their choosing, such as community engagement, curriculum development, and research (Table 2).

### 3.3 Theme 3: Variable Perceived Value Toward Promotion

Participants highlighted both obstacles and progress leveraging their DEI efforts, particularly those related to mentoring, toward academic promotion. One participant noted that these efforts do not appear to be valued in the same way as other scholarly work:

“Scholarly work and output that has to be done frequently might have to take a backseat to some of your other diversity efforts, your recruitment efforts, your retention efforts, your curricular efforts. And when it comes to the time for like promotion and tenure, you might see that those who work in the DEI space are not promoted as quickly as others because they may fall behind on some of that scholarly work that’s looked at within the institutions.” (Participant 1020, a minoritized individual with a departmental role).

Another participant noted an increased tendency to consider DEI efforts in promotion but a persistent lack of institutional funding for such efforts, even with departmental backing:

“They are shifting the promotion guidelines to really include a lot more DEI initiatives and mentoring specifically. I do think it’s a positive thing to see that things are shifting, but I think it kind of remains a challenge. Like even if you have a supportive department chair, it doesn’t mean you have a supportive dean or that there’s money coming from the School of Medicine or even the university itself to fund these initiatives (or) protected time for people who do the work. We’re still not there.” (Participant 1003, a minoritized individual with departmental, institutional, and national roles).

Mentors were described as crucial to teaching participants how they could translate DEI initiatives into scholarly products to drive promotion:

“I found support from others, outside of (my institution) that also thought this (DEI) work was important. So they supported me and helped me to get promoted and to move this work forward.” (Participant 1006, a minoritized individual with departmental and national roles).

## 4 Limitations

Our study was limited to DEI leaders in academic EM; therefore, our findings are specialty-specific. However, many of the processes and structures surrounding leadership, advancement, and promotion are shared among other academic specialties. Participant leadership positions varied greatly (eg, committee chair vs vice chair, departmental vs institutional), which limits our ability to comment on any one specific role. Nevertheless, our goal was to investigate the pathways to DEI leadership roles rather than the roles themselves. Although it is possible that framing the broader study during recruitment impacted participant responses in this report, we began all interviews with broad, open-ended questions about pathways to DEI leadership positions and did not specifically probe other topics until the latter half of the interview. Finally, we acknowledge the possibility of investigator bias, which we attempted to mitigate by creating a diverse study team and deriving themes through consensus.

## 5 Discussion

To our knowledge, this is the first national qualitative study that investigates pathways to DEI leadership roles among current DEI leaders in academic EM. Our study reveals that academic EM faculty members’ pathways to DEI leadership roles are nonlinear, with undefined roles and expectations and variable perceived value toward academic promotion. Our findings are consistent with a prior study identifying a lack of evidence-based standards to guide the work of DEI leaders and a mismatch between institutional investments and directives,^12^ which may partially explain the largely self-directed initiatives and challenges leveraging DEI efforts toward promotion highlighted by the participants in our study. However, our work probes deeper into how DEI leaders acquired their roles in the first place, revealing that although the majority either informally assumed or were volunteered for their roles by administrators, others created their own positions.

Our study has several important implications for the development and support of DEI leaders within academic EM. First, establishing formalized pathways to DEI leadership—for example, by creating fellowship or other training opportunities as is done with education^13^ and administration^14^—might help better identify and attract those with an interest in advancing DEI initiatives while avoiding having to volunteer specific individuals for these roles. The latter dynamic between administrators and faculty risks exacerbating the minority tax, which refers to the additional and disproportionate burden placed on minoritized individuals to take on DEI responsibilities.^10^ Second, establishing DEI leadership roles requires resources. Providing financial support and protected time for DEI leaders can make DEI roles more attractive and feasible to assume. Third, the perception of variable value toward promotion suggests the need to innovate around DEI leaders’ career advancement. Although DEI activities may not result in conventional scholarly products (eg, publications), they may nevertheless produce equally valuable outputs, such as greater workforce diversity,^15^ which could also be considered in the institutional promotion process. Such recognition would not only ensure these positions are valued within the academic hierarchy but may also ameliorate the underrepresentation of minoritized individuals in general leadership roles and higher academic ranks.^16^

Study participants also underscored the self-driven nature of DEI initiatives fueled by a sense of moral obligation. This sentiment—coupled with being voluntold for roles and minimal institutional support^12^—highlights a need for systematic changes. Legislative changes and societal polarization^17^ make solidifying career pathways, developing well-resourced roles, and weaving DEI efforts into the broader institutional fabric even more critical. Such measures are essential not only for supporting the individuals in these roles but also for ensuring the sustainability and effectiveness of DEI initiatives at large.

Overall, our study lends important insights into the pathways to DEI leadership in academic EM while also spotlighting resourcing and promotion as areas of opportunity for academic institutions to support DEI leaders. By addressing these issues, academic institutions can enhance their DEI efforts (in EM and beyond), improving academic diversity and health equity.

## Author Contributions

E.O. and A.N.C. conceptualized the study. A.N.C. and B.T. performed data collection. M.F.M., A.B., B.T., D.O., and A.N.C. analyzed the data, with E.O. and A.N.C. performing a critical review and evaluation of the results. M.F.M. primarily authored the paper, with all authors conducting final reviews and edits.

## Funding and Support

This work was supported in part by the Center for Innovations in Quality, Effectiveness, and Safety (#CIN 13-413).

## Conflict of Interest

All authors have affirmed they have no conflicts of interest to declare.

## References

[1] Parast L., Mathews M., Martino S., Lehrman W.G., Stark D., Elliott M.N. (2022). Racial/ethnic differences in emergency department utilization and experience. J Gen Intern Med.

[2] Hong L., Page S.E. (2004). Groups of diverse problem solvers can outperform groups of high-ability problem solvers. Proc Natl Acad Sci U S A.

[3] Takeshita J., Wang S., Loren A.W. (2020). Association of racial/ethnic and gender concordance between patients and physicians with patient experience ratings. JAMA Netw Open.

[4] Persky S., Kaphingst K.A., Allen V.C., Senay I. (2013). Effects of patient-provider race concordance and smoking status on lung cancer risk perception accuracy among African Americans. Ann Behav Med.

[5] Nguyen A.M., Siman N., Barry M. (2020). Patient-physician race/ethnicity concordance improves adherence to cardiovascular disease guidelines. Health Serv Res.

[6] Tsuchida R.E., Mbele N., Chopra Z. (2024). Identifying the prevalence and characteristics of diversity, equity, and inclusion leaders in academic emergency medicine. AEM Educ Train.

[7] Tong A., Sainsbury P., Craig J. (2007). Consolidated criteria for reporting qualitative research (COREQ): a 32-item checklist for interviews and focus groups. Int J Qual Health.

[8] Campbell K.M., Rodríguez J.E. (2019). Addressing the minority tax: perspectives from two diversity leaders on building minority faculty success in academic medicine. Acad Med.

[9] Campbell K.M. (2021). The diversity efforts disparity in academic medicine. Int J Environ Res Public Health.

[10] Rodríguez J.E., Campbell K.M., Pololi L.H. (2015). Addressing disparities in academic medicine: what of the minority tax?. BMC Med Educ.

[11] Bernard H.R. (2006).

[12] Esparza C.J., Simon M., London M.R., Bath E., Ko M. (2024). Experiences of leaders in diversity, equity, and inclusion in us academic health centers. JAMA Netw Open.

[13] Jordan J., Ahn J., Diller D. (2021). Outcome assessment of medical education fellowships in emergency medicine. AEM Educ Train.

[14] Jarou Z. EMRA fellowship guide: administration/ED operations/ patient safety & quality improvement, 2nd edition. https://www.emra.org/books/fellowship-guide-book-2018/2-admined-opspatient-safety.

[15] Garrick J.F., Perez B., Anaebere T.C., Craine P., Lyons C., Lee T. (2019). The diversity snowball effect: the quest to increase diversity in emergency medicine: a case study of Highland’s Emergency Medicine Residency Program. Ann Emerg Med.

[16] Linden J.A., Baird J., Madsen T.E. (2022). Diversity of leadership in academic emergency medicine: are we making progress?. Am J Emerg Med.

[17] Saha S. (2023). After affirmative action — working toward equitable representation in medicine. N Engl J Med.

