# Supplementary material for: Navigating Nonlinear Pathways: Challenges and Opportunities for Diversity, Equity, and Inclusion Leaders in Academic Emergency Medicine
Source: J Am Coll Emerg Physicians Open. 2025 Feb 14;6(2):100060. doi: 10.1016/j.acepjo.2025.100060 (PMC11874557; doi:10.1016/j.acepjo.2025.100060)
Supplement: Supplementary Material [file mmc1.docx]

**Supplementary Appendix 1. Recruitment Script**

Dear Potential Research Subject:

We are conducting a research study titled "Faculty Perceptions and Experiences of Diversity Efforts in Academic Emergency Medicine” led by Dr. Edgardo Ordoñez, Principal Investigator (Baylor College of Medicine) and Dr. Anita Chary, Co-Investigator (Baylor College of Medicine). The purpose of this study is to describe the perceptions of the diversity tax amongst academic emergency physicians and investigate whether participants feel an increased burden of this tax based on individual or intersectional identities. We are contacting you, because as an ED physician leader that is directly involved in diversity, equity, and inclusion (DEI), your firsthand experience with this topic is valuable. We are identifying potential participants in many ways - including through emergency medicine organizations and direct referrals – and anticipate interviewing up to 25 individuals for this project.

If you agree to participate in the study, we will schedule a videoconference call with you to learn about your program. Interviews will last approximately 20 to 30 minutes. We will ask you general questions about DEI work in your ED and your perspectives and experiences about the minority/diversity tax in EDs. We will not be gathering any information about patients during the interview. We will take all steps possible to keep this information confidential.

Participation in the study is voluntary. You decide whether you want to take part of not. If you do not take part, you will lose none of your benefits or rights. You may decide to stop taking part at any time.

Additionally, you may stop the interview and/or withdrawal from the study at any time during the interview. It will not cost you to take part in this study. Participants will receive a $50 gift card upon completion of participation. If you wish to withdraw from the study after study participation, you request this by contacting the principal investigator Dr. Anita Chary at 713-791-1414 or by email at [anita.chary@bcm.edu](mailto:anita.chary@bcm.edu). If we have not yet deleted the link between your identity and your interview transcript, then we will delete the transcript from your interview. This research study does not have a sponsor. It is being funded by the Baylor College of Medicine Department of Emergency Medicine.

If you have any questions about this study, please contact Dr. Chary at 713-791-1414 or by email at [anita.chary@bcm.edu](mailto:anita.chary@bcm.edu). The phone number may be answered by an assistant who will forward the message. If you have questions about your rights as a research subject, contact the Institutional Review Board for Human Subject Research for Baylor College of Medicine & Affiliated Hospitals at (713) 798-6970.

Thank you for your time.

**Supplementary Appendix 2. COREQ (COnsolidated criteria for REporting Qualitative research) Checklist**

A checklist of items that should be included in reports of qualitative research

| **Topic** | **Item No.** | **Guide Questions/ Description** | **Author Responses** |
| --- | --- | --- | --- |
| **Domain 1: Research team and reflexivity** | | | |
| *Personal Characteristics* | | | |
| Interviewer/facilitator | 1 | Which author/s conducted the interview or focus group? | Interviews were conducted by BT and VR |
| Credentials | 2 | What were the researcher’s credentials? E.g. PhD, MD | BT: MPH  VR: MPH |
| Occupation | 3 | What was their occupation at the time of the study? | Research assistant |
| Gender | 4 | Was the researcher male or female? | VR, BT: Female |
| Experience and training | 5 | What experience or training did the researcher have? | Both interviewers held an MPH and were trained in qualitative interviewing by AC, the senior author and an expert in qualitative methodology who holds a PhD in Anthropology. AC received formal doctoral training in qualitative methods. |
| *Relationship with participants* | | | |
| Relationship established | 6 | Was a relationship established prior to study commencement? | There was no relationship between participant and interviewer before the study. |
| Participant knowledge of the interviewer | 7 | What did the participants know about the researcher? e.g. personal goals, reasons for doing the research | Participants were informed that BT and VR were research assistants. |
| Interviewer characteristics | 8 | What characteristics were reported about the inter-viewer/facilitator? e.g. Bias, assumptions, reasons and interests in the research topic | Research assistants had no prior affiliation with any of the participants or the research topic. |
| **Domain 2: Study design** | | | |
| *Theoretical framework* | | | |
| Methodological orientation and Theory | 9 | What methodological orientation was stated to underpin the study? e.g. grounded theory, discourse analysis, ethnography, phenomenology, content analysis | Phenomenology |
| Sampling | 10 | How were participants selected? e.g. purposive, convenience, consecutive, snowball | Purposive sample of faculty sampled based on geographic region of the United States. Recruitment was based on a list of potential participants generated through a review of websites from ACGME-accredited EM residencies and supplemented by referrals from the Executive Committee of the Academy for Diversity and Inclusion in Emergency Medicine (Society for Academic Emergency Medicine). |
| Method of approach | 11 | How were participants approached? e.g. face-to-face, telephone, mail, email | Email |
| Sample size | 12 | How many participants were in the study? | 21 |
| Non-participation | 13 | How many people refused to participate or dropped out? Reasons? | 35 individuals were approached but did not respond and as such did not complete an interview  4 individuals expressed interest but were unable to complete an interview due to lack of mutual availability with the interviewer |
| *Setting* | | | |
| Setting of data collection | 14 | Where was the data collected? e.g. home, clinic, workplace | Data were collected over the audiovisual conference service Zoom; audio was digitally recorded. |
| Presence of non-participants | 15 | Was anyone else present besides the participants and researchers? | No |
| Description of sample | 16 | What are the important characteristics of the sample? e.g. demographic data, date | Interviews were conducted from March-May 2023.  Demographics collected (race, ethnicity, gender, LGBTQ) are reported in Table 1. Interviewees were asked to self-report how they identified in each of these domains. All interviews were conducted in English.  Exact names of participants’ DEI leadership positions are not included, as this would make participants identifiable. |
| *Data collection* | | | |
| Interview guide | 17 | Were questions, prompts, guides provided by the authors? Was it pilot tested? | An interview guide with questions and prompts was developed and pilot tested within the research team |
| Repeat interviews | 18 | Were repeat interviews carried out? If yes, how many? | No |
| Audio/visual recording | 19 | Did the research use audio or visual recording to collect the data? | Audio recording |
| Field notes | 20 | Were field notes made during and/or after the interview or focus group? | No |
| Duration | 21 | What was the duration of the interviews or focus group? | The mean length of interviews was 37 minutes |
| Data saturation | 22 | Was data saturation discussed? | See Data Analysis section.  The researchers used a matrix in Excel to organize data by participant and label themes. No new themes emerged after 12 interviews |
| Transcripts returned | 23 | Were transcripts returned to participants for comment and/or corrections? | No |
| **Domain 3: analysis and findings** | | | |
| *Data analysis* | | | |
| Number of data coders | 24 | How many data coders coded the data? | All listed co-authors coded study data |
| Description of the coding tree | 25 | Did authors provide a description of the coding tree? | Data were coded regarding:   1. How the participant assumed their DEI leadership role 2. Participant’s discussion with their departmental/institutional leadership regarding role expectations 3. Activities undertaken through the DEI leadership role 4. Personal and professional impacts of the DEI leadership role |
| Derivation of themes | 26 | Were themes identified in advance or derived from the data? | Themes were derived from the data using an inductive approach. |
| Software | 27 | What software, if applicable, was used to manage the data? | Microsoft Excel |
| Participant checking | 28 | Did participants provide feedback on the findings? | No |
| *Reporting* | | | |
| Quotations presented | 29 | Were participant quotations presented to illustrate the themes/findings?  Was each quotation identified? e.g. participant number | Quotations are presented in the main text as well as in Table 2. Quotes are identified by participant number, and demographic characteristics of each participant are included. Specific titles of DEI leadership roles are not included, as these would allow identification of participants. |
| Data and findings consistent | 30 | Was there consistency between the data presented and the findings? | Yes |
| Clarity of major themes | 31 | Were major themes clearly presented in the findings? | Three major themes were identified and presented in the results: (1) non-linear pathways, (2) unclear expectations, (3) variable value for promotion |
| Clarity of minor themes | 32 | Is there a description of diverse cases or discussion of minor themes? | The greatest diversity of experience was described as related to theme 1, non-linear pathways to leadership roles; we highlight three distinct paths. We also describe a diversity of expectations of the role and mentorship experiences related to career promotion. |

Developed from: Tong A, Sainsbury P, Craig J. Consolidated criteria for reporting qualitative research (COREQ): a 32-item checklist for interviews and focus groups. *International Journal for Quality in Health Care*. 2007. Volume 19, Number 6: pp. 349 – 357

**Supplementary Appendix 3. Interview Guide with Academic Emergency Medicine DEI Leader**

Interview questions in black informed the current manuscript.

**Demographics**

1. What DEI leadership roles do you have within your emergency department?
2. How long have you held this position?
3. What DEI leadership roles do you have within your institution?
4. How long have you held this position?
5. Do you have leadership roles outside your institution—at the regional or national level?
6. If yes, what are those specific roles?
7. How long have you held that role?
8. Are you involved in any other responsibilities that you feel define your role in DEI?
9. How many years have you been in EM practice in total including residency?
10. How many years have you been in EM practice since (after) residency?
11. What is your current academic rank? (Instructor, Assistant, Associate, Full Professor)
12. Can you share how you identify racially?
13. How do you identify ethnically? Do you identify as Hispanic/Latinx?
14. What is your gender identity? (cis-man, cis-woman, trans-man, trans-woman, non-binary, other)
15. Can you share if you identify as LGBTQ+?
16. Are there any other identities you have that are important to defining who you are?  
    *Prompt:* This might include an identity like first-generation college graduate.

**Understanding Context and Rapport Building**

I’d now like to ask you some questions about your experiences of DEI work.

1. Can you tell me about your responsibilities and efforts related to DEI in your role in your emergency medicine department? 
   *Prompts:* These might include mentoring, recruitment, research, awareness campaigns, organizing or facilitating events that promote DEI.

*Ask all interviewees if they didn’t mention mentoring about their mentoring efforts.* 

1. How did you come to assume your DEI leadership role in your emergency medicine department?

1. How does your involvement in DEI-related work impact you?  
   Prompt: *You can tell me how it’s impacted you personally, professionally, positively, negatively, or anything you wish to share.*

*Alternative phrasing:* ***Follow-up with all interviewees if they haven’t already answered this question:*** 
Has your involvement in DEI work positively or negatively impacted your professional life? In what ways? 
*Sub-Prompts:* How supported do you feel in your department in your academic journey in general—not just as related to your DEI efforts?   How about in your promotion process?  What type of support might you like to achieve promotion?

1. How do you balance your DEI work with your clinical load and other scholarly work/academic interests?

*Sub-Prompts:*  
Do you have protected time to support your DEI work?  If you have protected time, do you feel this time is adequate to accomplish your DEI efforts?

1. How do you feel about your emergency department climate as it relates to DEI?  
   *Prompts:*  
   What level of resources and support do you have to carry out your work related to DEI? 
   Is it sufficient?

**Experiences/Examples of Minority/Diversity Tax**

I’d like to ask you now specifically about your experiences related to the minority tax. You’ve probably heard of this term. I want to define how we’re using it for the purposes of our study. The minority tax is the extra/additional burden of time, resources and requests asked of people with minoritized social identities to represent and advocate for their communities. We are using the term diversity tax because it is broader and includes social identities outside of those with minoritized racial/ethnic identities.

1. Do you feel like you personally have experienced the diversity tax? If so, how?
2. Beyond your personal experience, have there been times you have noticed a diversity tax in your academic department otherwise? If so, how so?
3. Generally, do you feel colleagues in your department are aware of the minority tax? I’m referring to your colleagues generally, and not just those holding minoritized social identities.

**Strategies to Address Diversity Tax**

1. Have there been any discussions with your department leadership about the diversity tax? If so, what have those discussions been like?   
   *Prompts:* 
   Who raised the discussion topic? Was it someone with a minoritized identity?  
   What type of language have those discussions employed about the concept of the diversity tax?  
   Have the discussions used the term “minority tax” or have you spoken more broadly about general issues related to the disproportionate burden placed on specific individuals?
2. What can be done to address the issue of the minority tax in academic departments of EM?  
   *[For all] If the participant hasn’t addressed this in their response, prompt with:*

Whose responsibility is it to address it?

1. What are some strategies you have used to address the diversity tax? In general? For yourself? For others?

Thank you for participating. Do you have anything to add?
